# A Multi-Channel, Flex-Rigid ECoG Microelectrode Array for Visual Cortical Interfacing

**DOI:** 10.3390/s150100832

**Published:** 2015-01-06

**Authors:** Elena Tolstosheeva, Víctor Gordillo-González, Volker Biefeld, Ludger Kempen, Sunita Mandon, Andreas K. Kreiter, Walter Lang

**Affiliations:** 1 Institute for Microsensors, Actuators and Systems (IMSAS), Microsystems Center Bremen (MCB), University of Bremen, Bremen 28359, Germany; E-Mails: vbiefeld@imsas.uni-bremen.de (V.B.); lkempen@imsas.uni-bremen.de (L.K.); wlang@imsas.uni-bremen.de (W.L.); 2 Institute for Brain Research, Center for Cognitive Sciences, University of Bremen, Bremen 28359, Germany; E-Mails: gordillo@brain.uni-bremen.de (V.G.-G.); mandon@brain.uni-bremen.de (S.M.); kreiter@brain.uni-bremen.de (A.K.K.)

**Keywords:** biomedical electrodes, implantable biomedical devices, *in vivo*, neural microtechnology, neural prosthesis

## Abstract

High-density electrocortical (ECoG) microelectrode arrays are promising signal-acquisition platforms for brain-computer interfaces envisioned, e.g., as high-performance communication solutions for paralyzed persons. We propose a multi-channel microelectrode array capable of recording ECoG field potentials with high spatial resolution. The proposed array is of a 150 mm^2^ total recording area; it has 124 circular electrodes (100, 300 and 500 μm in diameter) situated on the edges of concentric hexagons (min. 0.8 mm interdistance) and a skull-facing reference electrode (2.5 mm^2^ surface area). The array is processed as a free-standing device to enable monolithic integration of a rigid interposer, designed for soldering of fine-pitch SMD-connectors on a minimal assembly area. Electrochemical characterization revealed distinct impedance spectral bands for the 100, 300 and 500 μm-type electrodes, and for the array's own reference. Epidural recordings from the primary visual cortex (V1) of an awake Rhesus macaque showed natural electrophysiological signals and clear responses to standard visual stimulation. The ECoG electrodes of larger surface area recorded signals with greater spectral power in the gamma band, while the skull-facing reference electrode provided higher average gamma power spectral density (γPSD) than the common average referencing technique.

## 1. Introduction

Cortical electrode devices provide a deeper insight and understanding of how the brain processes information and therefore advance the performance of brain-computer interfaces (BCIs) and neuroprosthetics. In this regard, high-density electrocorticographic (ECoG) electrode arrays, capable of recording neural signals directly from the cortical surface, offer detailed information about cognitive behaviour with high speed and accuracy and therefore serve as a basis for weakly-invasive high-performance BCIs [1]. ECoG signals excel EEG in their signal quality: ECoG has higher spatial resolution (in the mm-range [2,3]) and signal-to-noise ratio [4,5]. Frequencies above 40 Hz are difficult to record in a reliable manner with EEG, while ECoG has the bandwidth to cover the broad gamma band from 40 to several hundred Hertz [6]. Both ECoG and intracortical recordings closely correlate with specific aspects of sensory-motor and cognitive function [2,7–15], as well as with the fMRI BOLD signal [16–18], and allow observation of interactions between distant cortical sites [19–21]. Although intracortical recordings can measure the neuronal activity of a single cortical cell, their functional stability is very limited over time [22–26]. For these reasons, ECoG recordings are an attractive alternative signal-acquisition method for the study of brain function and neuronal interactions, and for weakly invasive BCIs with the aim of improving the quality of life of people afflicted by neurological disorders [27–37].

Clinical ECoG applications include the localization of epileptic seizures, intraoperative functional mapping and assessment of the limits of resection. Typically, these electrodes are of 1 to 2.3 mm diameter and 10 mm inter-electrode distance, embedded in a 0.4–0.6 mm-thick Silastic^®^ base [23,38]. Smaller-area electrode arrays, for dedicated research in humans, are implanted together with the larger ones, and these have electrodes of 70–1500 μm diameter and 1–4 mm inter-electrode distance [39,40].

Current ECoG microelectrode arrays used in research are highly flexible [41] and only several microns thick; they have also reached much higher electrode count: up to 252 [42] and 360 [43], with typical electrode diameters ranging from 300 to 1000 μm. The effect of electrode size on the coherence of ECoG signals has already been studied, but only with electrodes larger than 1mm and for inter-electrode distances greater than 1 mm [44,45]. Therefore we aimed to provide an electrode array with electrodes of different size in close neighbourhood that will allow for future investigations on the effects of electrode size in the sub-millimetre range. The highly flexible nature of these ECoG microelectrode arrays has required non-conventional assembly techniques for electrical connectors such as zero-insertion-force (ZIF) connectors [46–49]; anisotropic conductive film (ACF), applied between the pad-end and a fan-out PCB [43,50–53]; soldering of through-hole connectors directly to an ultra-flexible platform [42,54,55]; and isotropic conductive epoxy [56–58]. Each of these solutions can be advantageous within a certain range of electrode count and array application. High-electrode count ECoG arrays for applications on freely-moving animals call for miniature connectors, preferably fine-pitch and densely packed ones. The connector-assembly on these highly flexible devices can turn into a challenging task. A perfectly flat and rigid platform could solve this problem, provided it is monolithically integrated into the fabrication flow of the highly flexible device. Inspired by the F2R approach for the assembly of microsensors onto catheters [59], we made first advances toward a highly flexible ECoG array containing a rigid assembly platform [60,61]. In this paper we propose a finalized fabrication and assembly flow for a multi-channel ECoG microelectrode array, which bears a flat and rigid platform, cast out of the substrate wafer during microfabrication. We investigated the reliability of the assembly platform with electrical tests. Furthermore, we characterized the array electrodes (100, 300 and 500 μm in diameters) by means of electrochemical impedance spectroscopy and measured the effect of electrode size on impedance magnitude and phase. Finally, we demonstrated that the array worked *in vivo* via epidural recordings from the visual cortex of an awake Rhesus macaque. We showed its usability for investigating effects of electrode size on the recorded ECoG signals and for comparing the influence of a skull-facing reference electrode with a common average reference signal.

## 2. Materials and Methods

### 2.1. Array Design

**Figure 1.**
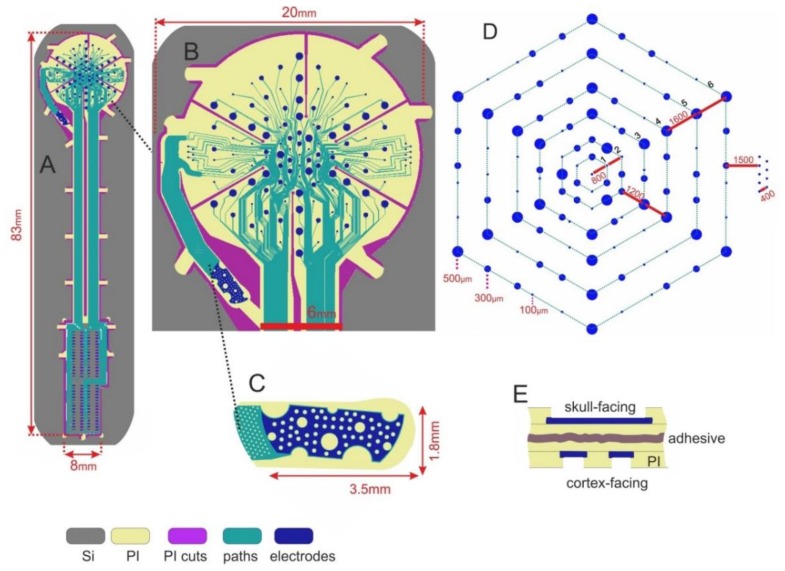
(**A**) ECoG electrode array; (**B**,**D**) Signal-acquisition area; (**C**) Reference electrode (top-view); (**E**) Reference electrode fixed to the array's backside (cross-section).

**Figure 2.**
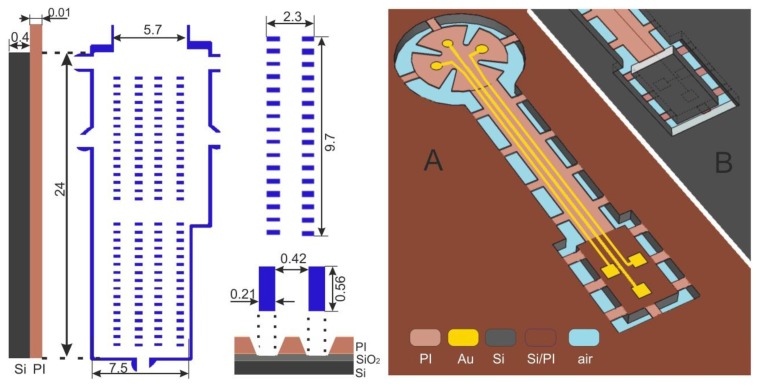
(**Left**) Connector-assembly region; (**Right**) A free-standing flex-rigid ECoG array.

The layout of the proposed ECoG electrode array is shown in Figure 1A: its electrodes are enclosed by a circular area, connected by a straight cable to a rectangular assembly region. The electrode and cable regions were designed to be embedded in polyimide (PI), while the assembly portion was to be made out of silicon (Si). The ECoG signal-acquisition area (Figure 1B) contains 124 circular electrodes of three different sizes and a much larger reference electrode, situated beside the array. Conduction lines (15 μm wide) connect the electrodes to the assembly region where the output connectors are to be placed. Most connection lines are forced to pass through the centre of the array at a 15 μm inter-line distance in order to allow several radial cuts. These radial cuts perforate the flexible material of the array so that upon implantation it can conform to the curved shape of the brain surface without formation of folds. Beside the radial cuts there are curvilinear cuts which define the edges of the array, the outlines of its reference electrode and the borders of several flexible straps, which will mechanically fix the array to its carrier wafer in the final fabrication step. A detailed view of the ECoG electrode arrangement is shown in Figure 1D. Circular electrodes (109 in total) are designed in three different sizes (100, 300 and 500 μm in diameter)*,* allowing to investigate how electrode size affects signal characteristics and, at a later stage, the features of stimulus-induced and stimulus-evoked field potentials. They are situated on a set of concentric hexagons and span the ECoG signal-acquisition area. Additionally, two sets of electrodes (100 μm in diameter) are arranged in groups outside the outermost hexagon, intended for high-resolution spatial frequency test measurements. The reference electrode, shown separately in Figure 1C, has a surface area of 2.5 mm^2^, and it is orders of magnitude larger than any other single ECoG electrode to ensure high signal-to-noise ratio. After completion of all manufacturing steps all flexible straps are cut through to release the ECoG device from its carrier wafer; the reference electrode is bent to the backside of the array, thus facing the skull during *in vivo* recordings (see Figure 1E). Holes of different sizes were designed in the reference electrode (1) to prevent crack formation in the metal film and (2) to minimize parasitic capacitive coupling since the hole-pattern of the reference electrode is a mirror image of the underlying electrode pattern. A detailed view of the connector-assembly region is presented in Figure 2, left image. Its 200 mm^2^ surface area is intended for the assembly of four 32-pin SMD-connectors. Each soldering pad has 210 μm × 560 μm area. The distance between two adjacent pads is 420 μm. The area of the connector- assembly region is 24 mm × 7.5 mm; the thickness of the silicon interposer is 400 μm.

Usually, ECoG electrode arrays are fabricated by spin-coating a flexible stack on a substrate wafer, which is peeled from the substrate in the final step to become a thin flexible device. In order to realize a rigid platform which is monolithically integrated in the microfabrication process of a flexible device, the substrate can instead come into use. By processing the backside of the carrier wafer, the substrate beneath the ECoG electrodes/cable is fully removed, while a silicon rectangle is cast out beneath the assembly area. In this manner, a free-standing flex-rigid device is realized as shown in Figure 2, right image: top side in A and backside in B.

### 2.2. Microfabrication

**Figure 3.**
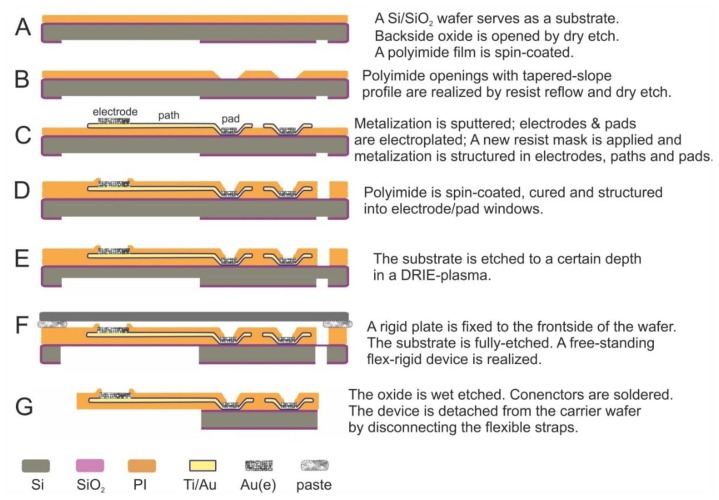
Microfabrication flow.

The fabrication of the free-standing flex-rigid device is presented in Figure 3. A 750 nm-thick thermal oxide is grown on a silicon wafer (4″, 380 μm thick). A photoresist mask, defining the array's flexible and rigid regions, is realized on the wafer backside (1.8 μm-thick AZ1518 photoresist; MA6 mask aligner, Süss, Garching, Germany; AZ726MIF developer, MicroChemicals, Ulm, Germany). The mask is transferred into the oxide beneath by dry etching (CF_4_-RIE plasma, ALCATEL 601E, ALCATEL, Annecy, France). The resist is stripped in AZ100 and the wafer is cleaned. Next, the wafer front side is spin-coated with VM651 organosilane adhesion promoter, followed by spin-coating of U-Varnish polyimide film at 3000 rpm for 40 s. The polyimide is soft-baked on a hotplate (5 min@80–120 °C & 5 min@120 °C) and then cured in a N_2_-atmosphere (10 min@450 °C peak), resulting in a final polyimide thickness of 5 μm. In this way, the backside oxide is structured into array outlines of flexible/rigid regions and then the first polyimide layer is realized on the wafer front-side (Figure 3A).

In the next stage, the wafer is cleaned by IPA, water-rinsed and dried. The polyimide surface is activated in O_2_ plasma to promote a good photoresist adhesion. A photoresist is spin-coated and structured into rectangular windows (10 μm-thick AZ9260 resist). The photoresist is then thermally reflowed to obtain a smooth step profile (1 h@120 °C). Then, the step profile of the reflowed resist is transferred in polyimide (RIE-plasma, STS ICP tool). The resist is stripped in AZ100. Hence, rectangular windows with a tapered slope are realized in polyimide; these windows define the regions where soldering pads will be situated (Figure 3B).

Next, the polyimide is dried, its surface is treated in O_2_ plasma and a 20 nm/300 nm-tick Ti/Au metallization stack is sputtered onto the polyimide. A photoresist film is applied and structured into electrode and pad windows (O_2_ plasma surface activation, 1.8 μm-thick AZ1518) and a 2.5 μm-thick gold layer is deposited via electroplating onto the windows. The photoresist is removed in AZ100. Another 1.8 μm-thick AZ1518 photoresist is spin-coated and structured into electrodes, paths and pads. The resist mask is transferred into the Au film (3–4 min in NaCN) and subsequently in Ti (30 s in BOE). The photoresist is stripped in AZ100. ECoG electrodes on polyimide and Ti/Au pads on Si/SiO_2_ are realized in this way (Figure 3C). The heat and O_2_ plasma treatment directly before metal sputtering on the polyimide layer should promote the adhesion between metal and polyimide.

In the following stage, VM651 organosilane adhesion promoter is applied and U-Varnish polyimide film is spin-coated, soft-baked and cured. A 10 μm-thick AZ9260 photoresist is spin-coated and structured into the arrays' outlines and cuts. This mask is transferred into polyimide in an O_2_ + CF_4_ RIE plasma and the photoresist is eventually stripped in AZ100. Another 10 μm-thick AZ9260 photoresist is applied, this time being structured as electrode and pad windows. These windows are then transferred in polyimide and the resist is stripped as described above. Hence, the array electrodes and pads are opened and the array outlines/cuts/straps are defined (Figures 1B and 3D). We structured the outlines/cuts/straps separately from the opening of electrodes/pads because of their different thicknesses: 10 μm and 5 μm, respectively.

In the final step, a 10 μm-thick AZ9260 photoresist is applied on the wafer backside. It is structured into a relatively large window defining the flexible area of the array and narrow lines surrounding the assembly area. The Si wafer is pre-etched on the backside to a 300 μm depth (80 min in SF_6_ + C_4_F_8_ DRIE plasma, STS ICP tool). The wafer is unloaded and an additional blank 4″ silicon wafer is fixed on its front side with a silicone paste (WLP 035, Fischer Elektronik, Lüdenscheid, Germany). The blank wafer serves as a mechanical stabilization platform, while the thermal-conductive paste provides a heat sink. The remaining Si is etched in *ca.* 40 min (Figure 3F), the blank wafer is detached and the paste is removed. The remaining oxide is then wet etched in BOE and the array wafer is rinsed and dried. In this way, a free-standing flexible electrode array with a monolithically integrated rigid interposer is fabricated. The array can be easily removed from its carrier wafer upon cutting the flexible straps (Figure 3G), which mechanically fix the array to its substrate.

### 2.3. Connector Assembly

The array's surface is activated in O_2_ plasma. A Sn-58Bi solder paste, with an 139 °C eutecticum and 20–38 μm solder powder size (LFM 65W, Almit GmbH, Michelstadt, Germany), is manually dispensed on the 128 array pads with a dispensing tip (250 μm inner-diameter) using a fluid dispenser under pressure (Nordson EFD, 1000 DVE). Four 32-pin SMD-type fine-pitch connectors (Omnetics, Nano Strip Series, NPD type, A71350-001, Omnetics Connector Corporation, Minneapolis, MN, USA) are aligned and mounted onto the pads, using a multi-purpose die bonder (FINEPLACER^®^ pico ma, Finetech GmbH & Co., KG, Berlin, Germany). The Omnetics connectors are picked by a vacuum holder, aligned to the array pads by the overlay vision alignment system (beam splitter) and then placed onto the pads by a lever arm. A 5 mm thick Al block is used as a placeholder between the FINEPLACER hotplate and the interposer in order to focus the heat primarily to the assembly platform and to reduce the heat transfer to the fully flexible electrode regions. The soldering heat-profile consists of the following plateaus: 40 s@100 °C, 40 s@129 °C, 30 s@140 s and 20 s@165 °C, whereas the temperature refers to the connector-assembly surface. After soldering the flux residues are rinsed (Flux Remover, followed by IPA) and adhesives are applied around the connectors and interposer to ensure mechanical stability (UHU plus endfest 300, UHU GmbH, Bühl (Baden), Germany; followed by UV-cured DELO-KATIOBOND GE-680, DELO Industrie Klebstoffe, Windach, Germany). The ECoG array is released from its carrier wafer by manually cutting the polyimide straps using a scalpel.

### 2.4. Array Characterization

**Figure 4.**
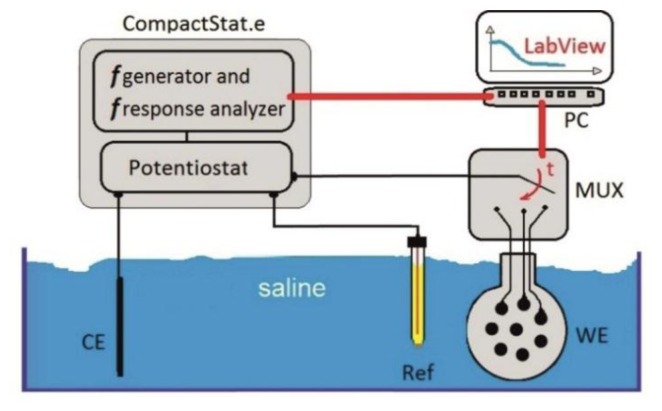
EIS test set-up with LabView-controlled multiplexer (MUX); *CE* stands for counter electrode, *Ref* for the external reference electrode and *WE* for the ECoG array.

The array was characterized by performing electrochemical impedance spectroscopy (EIS) in 0.9% saline *on its 128 electrodes* as shown in Figure 4. The electrodes were contacted via the four 32-pin Omnetics connectors of the array. A 2 cm^2^ Pt electrode served as counter electrode (CE) and an Ag/AgCl electrode (Ref) was the external reference (SensorTechnik Meinsberg GmbH, Meinsberg, Germany). A 10 mV sinus was applied at the Pt-electrode, while the frequency was swept from 10^5^ to 1 Hz. The electrode impedances were measured by a portable CompactStat.e impedance analyzer (IVIUM Technologies B.V., Eindhoven, The Netherlands). The switching between the electrode channels was performed by a NI-PXI 2530 multiplexer from National Instruments. An in-house developed LabView program provided the communication between the PXI and the CompactStat.

### 2.5. Implantation

All procedures and animal care were performed in accordance with the regulation for the welfare of experimental animals issued by the Federal Republic of Germany and were performed with the approval of the local authorities.

A six years old male rhesus monkey (*Macaca mulatta*), underwent implantation surgery in aseptic conditions. General anaesthesia was induced with ketamine/medetomidine (4 mg/kg/0.04 mg/kg injected intramuscularly) and maintained after intubation by ventilation with 30% O_2_–70% N_2_O air mixture plus 1.0% isoflurane, supplemented with fentanyl (3 μg/kg/h, intravenously). Atropine (0.05 mg/kg) was used to reduce salivation.

The ECoG array was implanted epidurally over the primary visual cortex (V1) on the brain's left hemisphere, covering the cortical map of the lower right quadrant of the visual field, between 1 and 5 degrees of eccentricity. We chose V1 because it is sufficiently large for our array to cover the representation of a lower quadrant of the visual field up to 5–6° in which our stimuli would be placed while also avoiding the centre of the visual field representation (fovea), where minimal eye movements would result in particular large shifts of activated regions. Localization of V1 in stereotactic coordinates was supported by anatomical MRI scans performed beforehand. A craniotomy of approximately 1.5 × 1.5 cm was performed using a piezo-surgery instrument (Piezosurgery^®^, mectron Deutschland Vertriebs GmbH, Köln, Germany), and the resulting boneflap was later refastened to the skull with a titanium stripe and screws. The cleft between the boneflap and the skull was sealed with calcium phosphate bone substitute (Norian™ CRS™ Fast Set Putty™, Norian Corporation, West Chester, PA, USA).

The connector-platform of our ECoG array was fixed to the surface of the acrylic cap with epoxy glue (Loctite 3090, Henkel, Düsseldorf, Germany). The connectors were protected from humidity and mechanical strain by a custom-made titanium chamber with an aluminium lid (NAN Instruments Ltd., Nazareth, Israel). The whole arrangement was fixed to the animal's head with dental acrylic (Palamed and Paladur, Heraeus Kulzer, Hanau, Germany) and titanium screws (Synthes GmbH, Solothurn, Switzerland) over a basic layer of UV-sensitive ceramic cement (Solid Bond P/S and Charisma Flow, Heraeus Kulzer).

### 2.6. Demonstration of Functioning in Vivo

**Figure 5.**
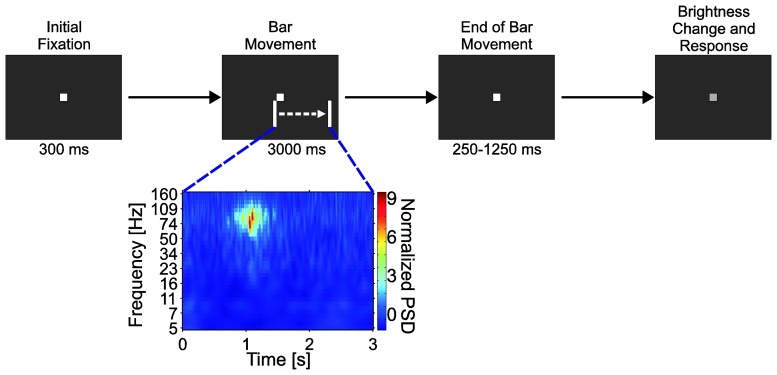
Receptive field mapping task. Movement of the bar stimulus over the receptive field of an electrode is reflected as a temporary increase in ECoG signal power.

Before array implantation, the animal was trained to maintain fixation on a small bright square on the centre of a 22-inch cathode ray tube display (100 Hz, 1152 × 864 [1024 × 768], NEC MultiSync FP1355, Tokyo, Japan) over a dark background, while ignoring any other stimulus in the screen. The distance between the screen and the monkey's eyes was 86 cm, and the animal sat on a custom-made primate chair with its head fixed in space. After an initial fixation period, a bright white bar (100 mm × 3 mm; 6.7° × 0.2° visual angle) moved in a direction perpendicular to its long axis across the lower right visual field (Figure 5). Twelve equiangular directions (0°–330°) corresponding to six different bar orientations were used in the experiment. The bars moved for three seconds with constant speed of 50 mm/s (3.33°/s). Upon the end of movement, the bar stimulus disappeared, followed by a variable interval (250–1250 ms) in which no stimulus besides the fixation point was present. Finally, the monkey had to respond to a small change in the brightness of the central fixation spot to receive a liquid reward. If the animal broke fixation (fixation window diameter 1.3°, centred on the fixation point) or responded too early or too late, the trial was aborted without reward.

The four Omnetics array connectors sent the raw signals via 3-inch cables (A71273-001, Omnetics Connector Corporation) to an adaptor board that interfaced the array to the recording system. Four 32-channel headstage preamplifiers (MPA32I, Multichannel Systems MCS GmbH, Reutlingen, Germany) amplified the ECoG signals ten times. Two 32-channel signal collectors (SC2 × 32, Multichannel Systems MCS GmbH, Reutlingen, Germany) fed the signals into programmable gain amplifiers (PGA64, Multichannel Systems MCS GmbH), with a gain of 100 for all channels. The amplified signals were then sent to the data acquisition system (USB-ME256, Multichannel Systems MCS GmbH). Signals were digitized at a sample rate of 25 kHz. In addition to the signals from the array electrodes, we recorded eye position information (ISCAN Incorporated, Woburn, MA, USA) and digital information about the timing of stimuli and task events. In-vivo data were acquired between one and three months after implantation. A recording session was used for the analysis only if every single stimulus was successfully presented at least ten times. A total of 525 successful trials recorded in eight sessions were used in the subsequent analyses.

Using custom routines developed in MATLAB^®^ (The Mathworks, Inc., Natick, MA, USA), the recorded data was filtered (direct-form FIR filter, order 422) between 1–150 Hz (gain 1, −3 dB at approx. 170 Hz) and down-sampled to 1 kHz. Forward and backward filtering was applied to avoid unwanted phase shifting. To analyse the ECoG signals in the time-frequency domain, the recorded data underwent wavelet transformation using complex Morlet wavelets with Gaussian shape in both time and frequency domains [10]. Power spectral density values (PSD) were calculated as the square of the absolute value of the wavelet-transformation divided by the Nyquist frequency (500 Hz). To build time-frequency plots of mean power spectral density, corresponding time-frequency bins of trials sharing the same stimulus were averaged and then normalized against the baseline taken from the initial fixation period of 300 ms before bar stimulus onset.

## 3. Results

### 3.1. Microfabrication

**Figure 6.**
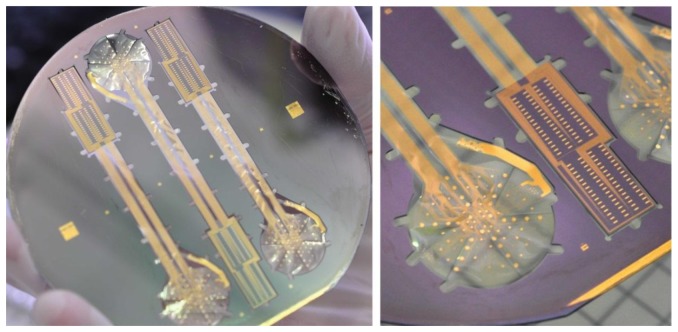
A 4″ wafer which contains three flex-rigid ECoG devices.

A fully processed wafer is shown in Figure 6. It contains three flex-rigid free-standing ECoG devices, spanned to the wafer by flexible straps; the signal-acquisition areas are highly flexible whereas the connector-assembly islands are rigid.

**Figure 7.**
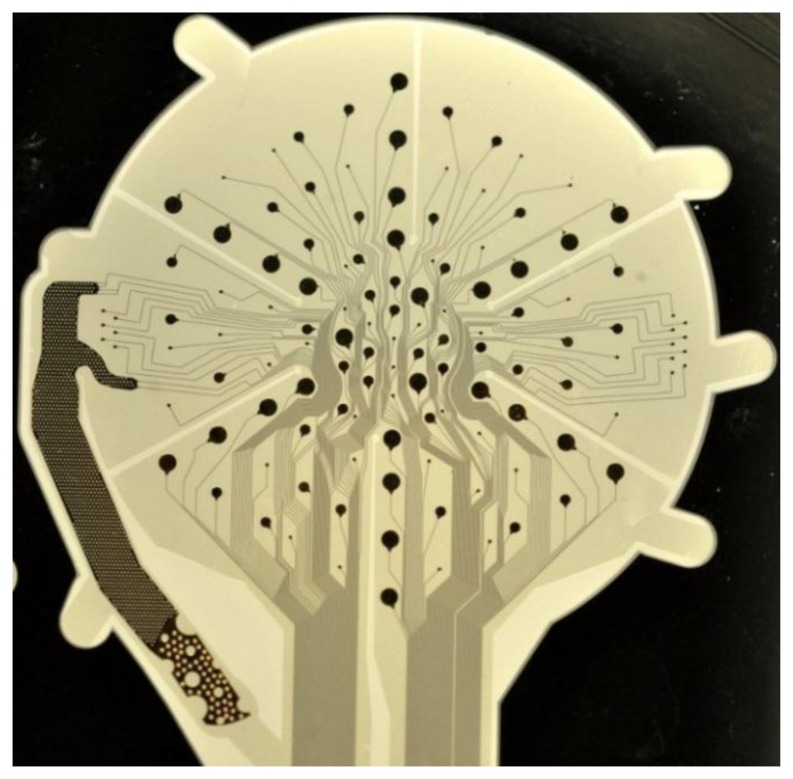
ECoG signal-acquisition area.

Figure 7 shows a magnification of a signal-acquisition area: 124 circular electrodes and the reference electrode placed beside them, which is connected to four separate metal paths. Hence, each of the four connectors has a separate connection path to the reference electrode.

**Figure 8.**
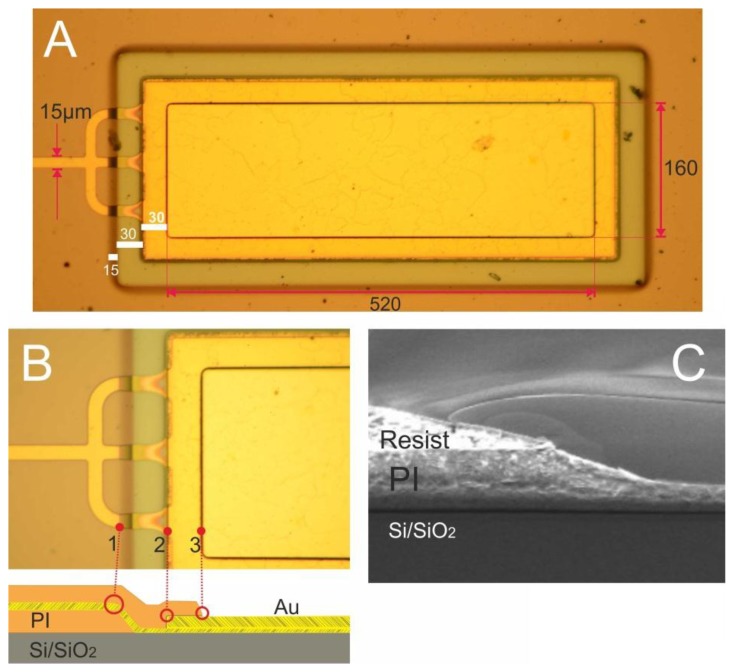
(**A**,**B**) Soldering pad; (**C**) Reflowed photoresist profile transferred to PI.

A top view of a soldering pad is presented in Figure 8A: it has a soldering area (polyimide opening) of 520 × 160 μm^2^ = 0.083 mm^2^; its metal area is slightly larger (30 μm wider on all sides) to prevent solder paste from diffusing into the metal paths. Figure 8B shows a magnification of the soldering pad: a 300 nm-thick metal path runs from left to right and splits into three sub-lines. These sub-lines run over the polyimide step (1), then over a Si/SiO_2_ region and enter a rectangular 2 μm-thick soldering pad (2); a top polyimide fully covers the metal paths and encircles the metal pad area (3). The sub-lines were designed to ensure that if one line breaks, there will be two others in reserve; the spacing between the lines was introduced because top PI adheres more strongly to the bottom PI than to Au.

Figure 8C shows the cross-section of a reflowed photoresist mask, whose profile was transferred into polyimide. The PI step has a slope of 20°, which can provide a smooth transition for a metallization film from the polyimide surface to the silicon substrate underneath. In this image the polyimide was etched only half-way to determine its slope angle and etch rate; in the final fabrication flow the PI is fully removed.

### 3.2. Connector Assembly

**Figure 9.**
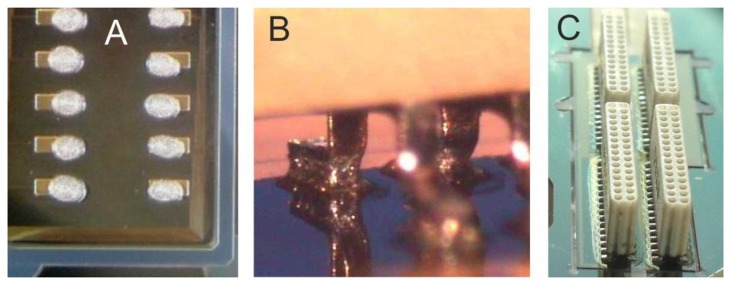
(**A**) Solder paste dispensed on pads; (**B**) Soldered connector leg; (**C**) Four soldered connectors after flux-clean.

**Figure 10.**
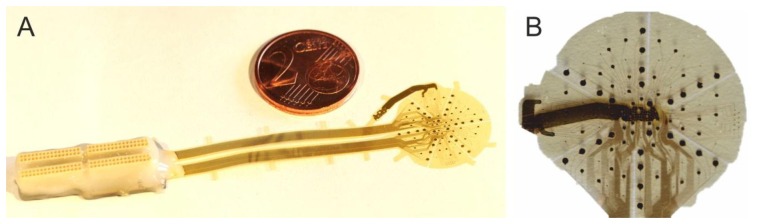
(**A**) ECoG array after wafer release; (**B**) The ECoG array with its skull-facing reference electrode.

The connector-assembly process is presented in Figure 9. The dispensing of solder paste was performed in a slightly off-pad fashion to ease the following connector alignment (A); alignment and positioning of Omnetics connections on the solder-paste-covered pads was performed with a FINEPL*A*CER tool. Applying the corresponding soldering heat-profile transformed the solder-paste spheres into solder-joints (B); four Omnetics connectors were soldered per array and the flux residues were cleaned (C); a packaged ECoG array with a fully encapsulated connector-assembly region can be seen in Figure 10A. Prior to implantation the bottom end of the reference electrode was flipped to the array backside and fixed by a biocompatible, high-temperature and moisture-resistant adhesive (Polytec EP653, Polytec PT GmbH, Waldbronn, Germany) to realize a skull-facing reference (Figure 10B).

### 3.3. Solder-Joint Characterization

**Figure 11.**
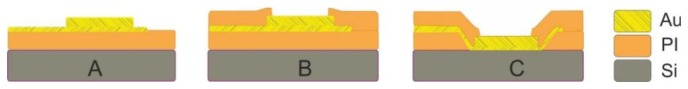
Specimen types used in adhesion (**A**–**C**) and electrical (**B**,**C**) tests.

The quality of solder-joints between pads and connector legs (Figure 9B) was investigated by means of electrical tests. For the electrical characterization we prepared two types of array specimen. The first type contained fully rigid soldering pads (see Figure 11C) fabricated according to Section 2.2. The second type was fabricated with state-of-the-art ECoG array pads containing PI under the metal layer (see Figure 11B), deliberately skipping fabrication step B in Figure 3. Omnetics connectors (four 32-pin connectors per specimen type) were soldered to each specimen and packaged in epoxy glue (see Section 2.3). The electrical characterization included short- and open-connection tests. During the short-connection test the dry inter-electrode dc resistance was measured with a conventional electronic multimeter in the 200 MΩ range: it revealed no parasitic short-connections. The open-connection test was performed in saline (see Section 2.4): a high number of malfunctioning solder-joints in B-type specimens were counted (15–27 open connections per 32-pin connector), whereas the *C*-type provided much more reliable solder-joints (0–3 open connections per 32-pin connector).

Additionally, we tested the adhesion of the Ti/Au metal pads to the underlying substrate of three specimen types (two samples à 32-pads per type) as shown in Figure 11: (A) metal pads on PI; (B) metal pads on PI which had experienced high thermal stress (curing of second PI layer for 10 min@ 450 °C); (C) metal pads on Si/SiO_2_ which had also undergone the same thermal stress. Scotch-tape adhesion tests [62] were performed as follows: an adhesive tape was applied on the metal pad surface; the tape tail was folded at 180° and pulled off quickly; the adhesion was considered satisfactory if no metal material was removed from the pad area. These adhesion tests revealed that the metal-PI adhesion is satisfactory if the samples do not undergo a second PI curing (specimen A); the metal-PI adhesion degrades after the second curing (specimen B), whereas metal-Si/SiO_2_ adhesion remains stable even after the second PI-curing (specimen C).

### 3.4. Electrode Characterization in Saline

**Figure 12.**
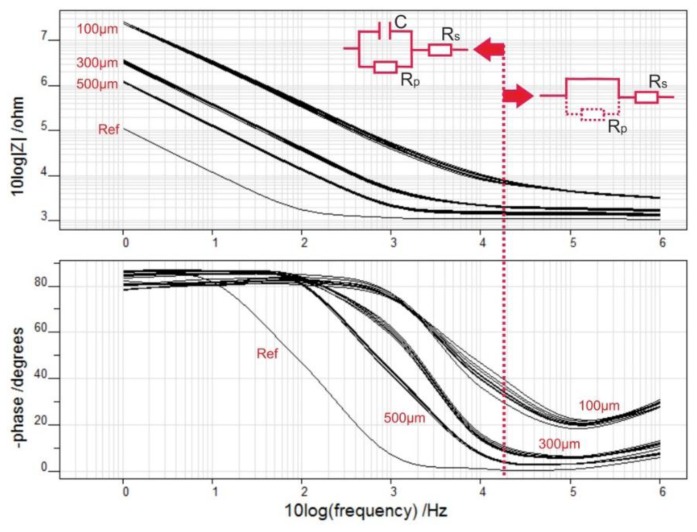
Impedance spectral bands according to electrode type; circuit diagrams.

**Figure 13.**
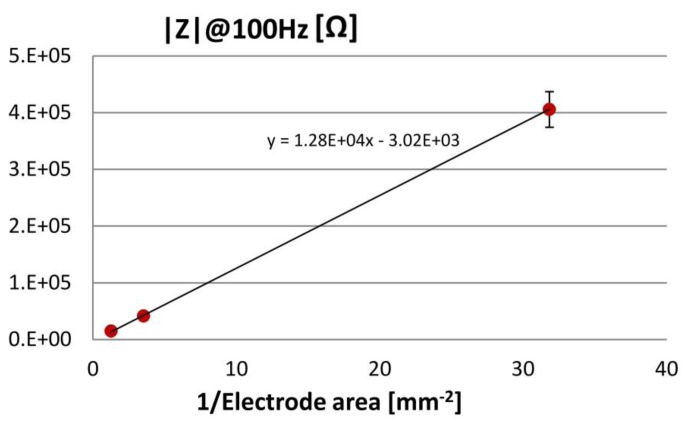
Impedance magnitude values at 100 Hz derived from the data of Figure 12, plotted against the inverse values of the electrode area (mean ± SD).

ECoG electrode arrays with fully rigid soldering pads (Figure 11C) were characterized by electrochemical impedance spectroscopy. The impedance spectral bands (magnitude and phase) were measured for each electrode type, all belonging to a common Omnetics connector (see Figure 12): the impedance magnitudes |Z| decrease with rising frequency, show capacitive behaviour in the low frequency range and resistive behaviour above 10 kHz. At 100 Hz, a frequency value associated with gamma ECoG activity, the average |Z| values are plotted in Figure 13 and they are inversely proportional to the surface areas of the 100, 300 and 500 μm-diameter electrodes.

Additionally, the impedance magnitude of the reference electrode at 100 Hz is eight times smaller than the impedance magnitude of the largest circular electrodes (500 μm-type).

The impedance of an electrode immersed in electrolyte can be modelled by an equivalent electric circuit X_c_‖R_p_+R_s_, with X_c_ being a capacitive reactance, both R_p_ and R_s_ ohmic resistances [63]. As the metal electrode is negatively charged with respect to electrolyte solution, a double layer is formed at the electrode-electrolyte interface, which resembles a charged capacitor. Its capacitance C is related to X_c_ by the angular frequency ω: C = (ωX_c_)^−1^. Additionally, as current passes via the electrode-electrolyte interface, the resistance R_p_ accounts for the associated charge transfer, whereas R_s_ sums up the electrolyte and array-path resistances [63].

**Figure 14.**
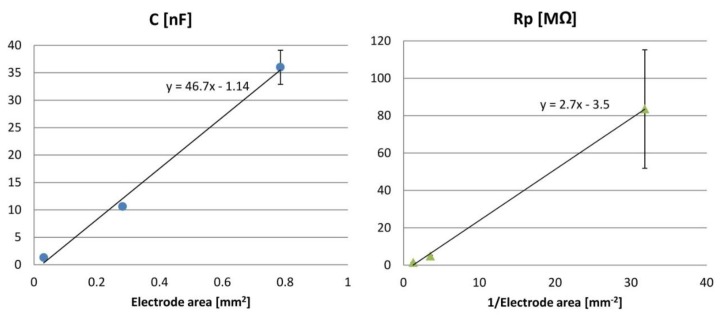
Values derived from Table 1 for the double layer capacitance *C* and charge-transfer resistance *R_p_* plotted against the electrode area.

Applying the above electric circuit model to the impedance data reveals that in the low frequency range C and R_p_ determine the steady impedance increase at frequencies; R_s_ accounts for the low ohmic behaviour above 10 kHz because C shunts R_p_ (see Figure 12, circuit diagrams). Furthermore, the impedance data of the ECoG array were fitted using IviumSoft Electrochemistry Software (Ivium Technologies B.V., The Netherlands) to derive the C, R_p_ and R_s_ values (mean + s.e.m.). As presented in Table 1 and Figure 14, R_s_ belongs to the low kΩ-range; R_p_ spans the 1–80 MΩ-range, and it rises linearly with decreasing electrode area; C is in the 1–36 nF range and increases linearly with electrode area.

### 3.5. Demonstration of Functioning in Vivo

**Figure 15.**
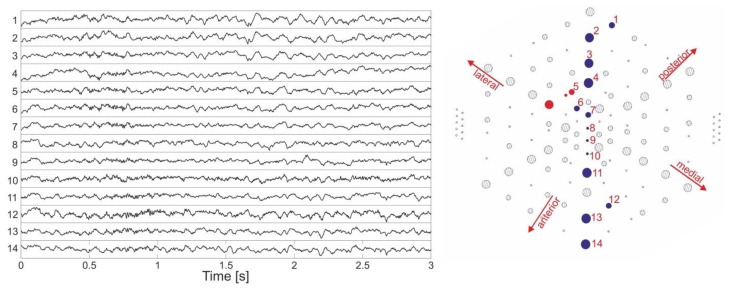
Electrophysiological signals (left image) recorded from electrode sites distributed over the array diameter (right image, #1–14).

The functioning of the ECoG electrode array was demonstrated *in vivo* while a Rhesus monkey performed a visual fixation task, and at the same time ignored bright bars moving across the bottom-right visual quadrant of a dark screen (see Section 2.6). Figure 15 shows example traces recorded from primary visual cortex (V1) of an animal six weeks after implantation. These signals were acquired during a 3 s-long instance of bar movement (left image) from 14 ECoG electrodes spanning the arrays' diameter (#1–14, Figure 15, right image). Distinct responses in the form of faster oscillations of the electrocorticographic signal were induced by the moving bar within the first second of stimulus presentation. Electrodes situated in proximity to each other clearly showed similar oscillatory responses at approximately the same time, while those of more distant electrodes occurred in more distinct epochs as expected because of V1's retinotopic visual field map. This further confirmed that changes in the ECoG signal corresponded to a response to the moving stimulus activating successively neighbouring regions of area V1.

**Figure 16.**
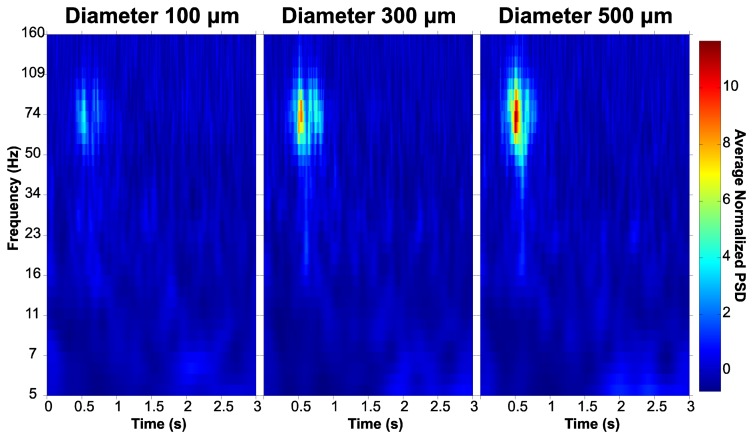
Time-frequency plots of normalized PSD for three neighbouring electrodes of different sizes situated in the middle of the array layout (red circles in the right image of Figure 15).

Figure 16 presents time-frequency plots of normalized power spectral density (PSD) from three neighbouring electrodes of different sizes (red dots in Figure 15). The data were averaged over all trials within a session presenting a specific moving bar stimulus (direction 300°), with stimulus onset at time zero. As shown in the figure, a rise in spectral power density can be recognized as the electrode diameter increases from 100, through 300 to 500 μm diameter. This increase in spectral power density was induced by the bar movement within the first second, with peak values situated at ca. 0.5 s for each of the three different electrodes; it was several times stronger than baseline and covered the 40–150 Hz frequency range at the maximum, with decreasing frequency span for smaller electrodes.

**Figure 17.**
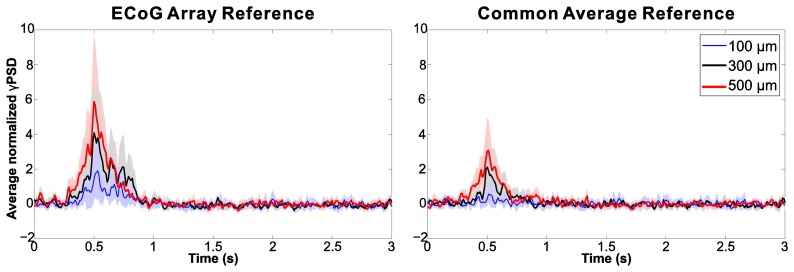
Time course of normalized gamma-PSD ± s.d. derived from time-frequency plots (shown in Figure 16), using a skull-facing reference electrode (**Left**) and common average referencing (**Right**).

To compare PSD values, 11 triplets of neighbouring electrodes were selected and measured over eight sessions (88 samples in total); the frequency bins between 40 and 150 Hz in the time-frequency matrix were averaged for each electrode to build time courses of average gamma-PSD (γPSD). Figure 17 presents for the three example electrodes the γPSD-time courses obtained with two different referencing methods: the usage of a skull-facing electrode resulted in an almost twofold increase in power value (left image), as compared to common average referencing [64,65] (right image). With respect to γPSD for both referencing techniques the smallest electrodes consistently recorded the smallest spectral power, while the largest electrodes recorded the greatest average γPSD in 71.6% (63/88) of the measurement triplets examined.

To complement the analysis of average PSD values, gamma-band signal-to-noise ratio (SNR_γ_) measurements were performed and then compared across electrode sizes. SNR_γ_ was measured as the ratio between the mean area beneath the full width at half maximum of the gamma PSD time courses and the standard deviation of the areas, measured over all successful trials in a session displaying the same stimulus. There was no statistically significant difference in SNR_γ_ between neighbouring electrodes of different sizes, χ^2^(2) = 5.1839, *p* = 0.0749 (Friedman; *n* = 88; Medians: [100 μm: 3.43, 300 μm: 2.87, 500 μm: 2.80]).

## 4. Discussion

In this paper we presented the design of an ECoG electrode array with high-electrode count and density. Electrodes of three different sizes (100, 300 and 500 μm in diameter) and a 2.5 mm^2^ skull-facing reference electrode were implemented to provide a device for testing their influence on the quality of ECoG signal-acquisition.

The high-electrode count and the array's application on freely-moving animals required a highly miniaturized connector-assembly platform. For this purpose, we developed a microfabrication flow which results in highly flexible 10 μm-thick polyimide (PI) bi-layer, with Ti/Au metallization in it and allows also the monolithic integration of a fully rigid interposer for the assembly of miniature SMD-type Omnetics connectors. The silicon interposer was fabricated via a two-step DRIE etching procedure.

State-of-the-art ECoG arrays are fabricated of polymer/metal/polymer, making use most often of either PI [42,47,49,50,54] or parylene [46,48,51] as flexible materials and of Pt [42] or Au [54,66] as metallization materials. In contrast to the presented device, state-of-the-art ECoG arrays are peeled from their wafer carriers and electrical connections are made by means of zero-insertion-force (ZIF) connectors [46–49], ACF on fan-out PCBs [43,50–53], soldering of through-hole Omnetics connectors [42] or isotropic conductive epoxy [56–58]. Each of the above assembly solutions is advantageous for a certain range of electrode count or array application. Nevertheless, if the focus is set on multi-channel ECoG arrays with connector-assembly area reduced to a minimum, then only soldering of through-hole Omnetics connectors [42] remains as a proposed connector-assembly technique. Compared to the work of [42], our fabrication process requires additional DRIE silicon etching, which increases fabrication time/costs/complexity. Nonetheless, exactly this additional fabrication step turns into a substantial advantage in terms of straightforward connector alignment and assembly and reliable solder-joints, requiring no additional rigid soldering jig to hold the array in place.

Electrical open and short-connection tests showed that fully rigid solder joints were much more reliable than those containing PI under the metallization pads. Moreover, adhesion tests revealed higher adhesion strength of the Si/SiO_2_-metal interface compared to the Si/SiO_2_/PI-metal interface, which most likely accounts for the higher reliability of the fully rigid solder joints. Comparing PI/metal adhesion before and after the second polyimide curing indicated a decrease in adhesion at the PI/metal interface, most likely caused by thermal stress exerted during the final curing step.

The array electrodes and its reference have impedance magnitudes that increase as the electrode surface area grows larger, as expected. The impedance magnitude of the reference electrode at 100 Hz is eight times smaller than the impedance of the largest circular electrodes, and the impedance at 100 Hz of the smallest electrodes is *ca.* 10^5^ times lower *than* the input impedance of a commercial headstage amplifier [42]. These impedance differences are of considerable importance for recording electrocorticograms with sufficiently high amplitude. Furthermore, the derived parameters of the equivalent electric circuit as suggested by [63] indicated a clear linear relationship between the capacitance and the electrode surface area, while the associated double-layer resistance rose linearly with the inverse of the electrode surface area. Compared to impedance values reported in literature, our measurements are equivalent to impedance values reported by Baek *et al.* (480 μm diameter) [54] and by Kim *et al.* (250 μm diameter) [66].

We designed this array layout to investigate the dependence between electrode properties and the characteristics of acquired signals for the rhesus macaque brain. This animal model was chosen because of the anatomical and physiological similarity between macaque and human brains. Furthermore, we could take advantage of the highly specific and well-documented spatial organization of the primary visual cortex, which allows recording stimulus-induced responses from different groups of electrodes according to the position of the visual stimulus shown to the animal. The array covers the cortical region representing visual field regions between one and five degrees of eccentricity, which simplifies the placement of visual stimuli and also facilitates array implantation.

To demonstrate the functionality of our design we implanted the array in a rhesus macaque and successfully recorded electrophysiological signals, including strong and distinct stimulus-induced responses, for several months. The temporal order in which the stimulus-induced responses occurred at the different electrodes was clearly related to the succession of positions traversed by the moving stimuli in the visual field. First recordings of our electrode array indicated that larger electrodes recorded stimulus responses with greater spectral power in the gamma band (40–150 Hz), distinctively stronger than the baseline signal, which can be attributed to the larger recorded population of neurons contributing to the stimulus-induced activity patterns. These *in vivo* results indicate that the ECoG array construction, design and fabrication were successful.

Furthermore, referencing the recordings to the skull-facing reference electrode provided signals with greater power than those obtained using a common average referencing technique. This difference can be explained by the synchronous activity in the gamma band induced over extended regions of area V1 by the long bar stimulus [67]. This activity is therefore acquired by a substantial subset of the array electrodes and therefore prominently contained in the common average reference which is obtained by averaging over all electrode signals. Subtracting this common average reduces the gamma band response in corresponding electrodes. Further advantages of integrating the reference electrode at the backside of the array are the avoidance of additional effort to place a separate reference during surgery; the saving of space, in particular if multiple arrays are implanted; and the minimization of spurious signals more likely to be picked up by a distant reference electrode facing the brain.

## 5. Conclusions/Outlook

A flex-rigid ECoG electrode array was proposed, which has high-electrode count and density. The highly flexible nature of the device allows bending from a flat into spherical shape without formation of folds, which matches the cortical surface. The highly planar and rigid connector platform (Si/SiO_2_), monolithically integrated in the structure of the flexible ECoG array, facilitates the assembly of miniature fine-pitch Omnetics connectors.

Furthermore, circular gold electrodes of three different sizes and high density were implemented in the array's signal-acquisition area. This will enable future investigations on the optimal size and density of ECoG electrodes with respect to different types of brain signals which provide different information content. Such knowledge will be helpful for design and configuration of dedicated brain computer interfaces for different specific purposes.

Epidural electrocorticographic recordings of strong and distinct stimulus-induced electrophysiological field potential signals from a Rhesus macaque's primary visual cortex (V1) demonstrated functioning of the proposed flex-rigid electrode array design. Larger electrodes recorded signals with greater spectral power in the gamma band (40–150 Hz). The use of a skull-facing reference electrode confirmed an expected higher spectral power as compared to the common average reference technique, distinctively stronger than the baseline.

These results support the advantage of engineering a flex-rigid electrode array with a monolithically integrated connector platform for the microfabrication of high-electrode count and density ECoG electrode arrays for neuroprosthetics and brain-computer interfacing applications.

## Figures and Tables

**Table 1.** Parameters of the electric circuit model (mean + s.e.m): *C* is the double-layer capacitance, *R_p_* the charge transfer resistance and *R_s_* the path + saline resistance.

**Electrode Diameter**	**Electrode Area [mm^2^]**	**R_s_ [kΩ]**	**R_p_ [MΩ]**	**C [nF]**
100 μm	0.031	2.6 ± 0.1	83.5 ± 31.7	1.3 ± 0.1
300 μm	0.283	1.8 ± 0.02	4.8 ± 0.4	10.6 ± 0.4
500 μm	0.783	1.5 ± 0.02	1.3 ± 0.1	36.0 ± 3.1
